# The Distribution of Innervation and Immune Cell Infiltration Is Different in Genital and Extragenital Variants of Lichen Sclerosus

**DOI:** 10.3390/biom12121767

**Published:** 2022-11-27

**Authors:** Dubravka Vuković, Marin Ogorevc, Ivo Tripković, Neira Puizina-Ivić, Mirna Saraga-Babić, Snježana Mardešić

**Affiliations:** 1 Department of Dermatovenerology, University Hospital Split, 21000 Split, Croatia; 2 Department of Anatomy, Histology and Embryology, University of Split School of Medicine, 21000 Split, Croatia; 3 Department of Plastic Surgery, University Hospital Split, 21000 Split, Croatia

**Keywords:** lichen sclerosus, vanilloid receptor, calcitonin gene-related peptide, mast cells, macrophages, pruritus

## Abstract

Lichen sclerosus (LS) is a progressive skin disease that is characterized by chronic inflammation of either genital or extragenital skin, and it disproportionately affects women. We analyzed the distribution of nerve fibers, vanilloid receptors, cell proliferation, mast cells and macrophages in genital and extragenital LS samples, as well as in healthy skin, by using immunohistochemistry. The total amount of intraepidermal nerve fibers was lower in LS samples compared to healthy controls, while the total amount of subepidermal nerve fibers and calcitonin gene-related peptide (CGRP) positive fibers was higher in genital LS samples compared to both extragenital LS and healthy controls. Cell proliferation, macrophage and mast cell density were increased in LS samples compared to healthy controls. Genital LS had a higher macrophage density compared to the extragenital variant. Mast cell distribution significantly differed between genital and extragenital LS samples, even though their total mast cell densities were similar. These findings could explain the differences between pruritic symptoms of genital and extragenital LS and provide targets for the research of novel therapeutic strategies for LS management.

## 1. Introduction

Lichen sclerosus (LS) is a chronic and progressive inflammatory skin disease that affects extragenital and genital skin [1]. It has two peaks of incidence, one during female prepubescence (approximately 7–15% of all LS cases) and the other in adulthood [2,3]. According to a recent study, the incidence of LS reaches 1.6% in women by the age of 80 years [4]. There are multiple clinical presentations of LS. It usually presents as pale, ivory-colored lesions with partially atrophic skin, distributed in a “figure of eight” in genital LS, however, it can also present as purpura, hyperkeratosis, sclerosis, fissures, ulcerations, blisters, scarring, and follicular plugging [5]. The main clinical symptoms of LS are itch (mainly in genital LS in females), soreness, dyspareunia or apareunia, urinary symptoms, and constipation (usually in children), or it can be asymptomatic [6]. About 20% of patients have extragenital lesions that favor the trunk and proximal extremities, accompanied by very mild symptoms of dryness and itch [1]. Vulvar lichen sclerosus leads to atrophy, destructive scarring, functional changes in the genital area, and in 5% of cases it may evolve to vulvar squamous cell carcinoma, and rarely into melanoma and basal cell carcinoma [1,7]. Genital LS shares histopathological features with the extragenital variant [8]. A band-like infiltrate of lymphocytes in the upper dermis, vacuolar degeneration of the epidermis basal layer, and mild homogenization of papillary dermis are seen in early lesions, while late lesions show orthokeratotic hyperkeratinization of both epidermis and upper dermis, hyalinization and sclerosis of the papillary dermis, vascular changes, telangiectasias and interstitial lymphocytic infiltrate [8]. It is diagnosed either by clinical appearance alone or after a biopsy [6]. Regarding therapy, first-line recommendations for genital LS are potent steroids in various regimens, while topical calcineurin inhibitors are used off-label as a common second-line local therapy option. If standard local therapy fails, acitretin, phototherapy, laser therapy, and surgery can be used. For extragenital LS, UVA 1 phototherapy, potent steroids, and topical calcipotriol are the treatments of choice [6].

Interaction between resident skin cells and skin neurons exists in many chronic inflammatory skin diseases, leading to an increased neurotrophin expression and proliferation of nerve fibers, called neurogenic inflammation of the skin [9]. It can be induced or enhanced by the release of substance P (SP) and calcitonin gene-related peptide (CGRP), and by the stimulation of transient receptor potential channels vanilloid 1 (TRPV1) and ankyrin 1 (TRPA1) [10].

CGRP is a vasodilatatory secretory peptide usually expressed in the cytoplasm of small-diameter neurons, as well as in large sensory neurons and motoneurons in the epidermis of vertebrates [11,12,13]. CGRP has a function in the pathophysiology of neuropathic and inflammatory pain, as it is expressed in polymodal nociceptors of viscera and skin [13]. In the skin, CGRP influences neurogenic vasodilatation, recruitment of inflammatory cells, shifting of Langerhans cells toward Th2 response, and activation of mast cells [9]. Psoriatic patients with pruritus have higher expression of receptors for CGRP compared to non-pruritic ones, while those with atopic dermatitis show skin with CGRP-positive nerve fibers [9].

TRPV1 is a nonselective cation channel that is activated by protons, noxious heat and some vanilloids, such as capsaicin [12]. It is expressed in the nociceptors and reacts to nociceptive stimuli, transduction of neuropathic pain and inflammation-provoked thermal hyperalgesia. TRPV1 expression has been found both in the sensory and motor neurons of humans and rats [12,13]. TRPV1 is expressed in sensory nerves and different non-neuronal cells of the skin, implying its role in the pathology of numerous pruritic skin disorders such as psoriasis, atopic dermatitis, and Netherton syndrome [10].

One of the best pan-neuronal markers is protein gene product 9.5 (PGP 9.5), also known as ubiquitin carboxyl-terminal hydrolase 1 (UCHL1) [14]. It is expressed in the cytoplasm of the majority of neurons [14,15] as well as in the nerve terminals and neuro-immune-endocrine cells of the skin. PGP 9.5 positive nerve fibers and dendritic cells have roles in chronic pruritic dermatoses, like atopic dermatitis and psoriasis [14,15,16]. Mast cells are present in all vascularized tissues [17] and their pathological activation can lead to chronic inflammation and pathogenic tissue remodeling [18]. Experimental animal models, as well as biopsies from atopic and psoriatic skin lesions, confirm reciprocal interaction between the sensory nerves and the mast cells containing secretory granules, which when released cause pruritus [19].

So far, studies have demonstrated overexpression of Ki-67 in genital lichen sclerosus compared to extragenital LS and healthy skin [20]. The inflammatory infiltrates in vulvar lichen specimens showed increased numbers of cells stained with monocyte/macrophage marker CD68 [21]. Tissue-resident macrophages and monocytes regulate tissue repair, regeneration, and fibrosis so their overexpression can lead to the hyperproduction of inflammatory factors [22]. Monoclonal antibody CD68 is therefore used to recognize tissue-resident macrophages in different tissues, including the skin [23].

Considering the difference in pruritus between genital and extragenital LS, we assume that the histopathologic features related to itch also vary between the two groups of lesions. The aim of this study, therefore, was to investigate the distribution of nerve fibers, vanilloid receptors, cell proliferation, mast cells, and macrophages in tissue samples of vulvar and extragenital LS, as well as healthy genital and extragenital skin. Additionally, we performed a co-localization analysis for VR1 (vanilloid receptor) with CGRP and tryptase to analyze whether VR1 is present in sensory nerve fibers and mast cells in LS lesions. Our findings could contribute to the further understanding of the role of neurogenic inflammation in the pathophysiology of LS and reveal potential new therapeutic targets for LS treatment.

## 2. Materials and Methods

### 2.1. Tissue Procurement and Processing

All protocols of this study were approved by the Ethical and Drug Committee of the University Hospital of Split (registry number: 2181-147/01/06/M.S.-22-02) and General Hospital of the Sibenik-Knin County (registry number: 01-23183/1-18), and were performed in accordance with the Declaration of Helsinki and other relevant regulations and guidelines. Skin biopsies were obtained from patients at the Department of Dermatoveneorology of the University Hospital of Split after they had given written informed consent. Samples were collected between January 2019 and December 2021 from a total of 40 female patients and separated into four groups: healthy extragenital skin, healthy genital skin, extragenital skin affected by lichen sclerosus, and genital skin affected by lichen sclerosus. Each group contained samples from 10 women, which is a sufficient samples size as calculated by Mead’s resource equation. Healthy extragenital and genital skin samples were obtained from surrounding tissue in biopsies of unrelated issues, usually the removal of moles (naevi) on the trunk and vulva. Samples of both extragenital and genital skin affected by LS were taken as a part of routine care at our institution—all lesions with the clinical features of LS are biopsied at first presentation, before any therapy is administered, to confirm the diagnosis. Pediatric patients presenting with LS are rarely ever biopsied, therefore, they were not included in our study. All extragenital samples (healthy and LS) were taken from the trunk, where extragenital LS lesions usually present. Information about all patients’ age was gathered, as well as the time from disease onset and the level of itch measured with a visual analog scale (VAS) for patients with LS. Tissues were processed as we have previously described [24]. Briefly, the tissues were fixed in 4% paraformaldehyde in phosphate-buffered saline (PBS), dehydrated in graded ethanol solutions up to 100%, paraffin-embedded, serially cut as 5 µm thick sections, and mounted on glass slides. For each biopsy, one section was stained with H & E (hematoxylin and eosin) and examined on an Olympus microscope (BX40; Tokyo, Japan) to confirm tissue preservation. H & E images were captured using CellA Imaging Software for Life Sciences Microscopy (Olympus, Tokyo, Japan).

### 2.2. Immunofluorescence Staining

Immunofluorescence staining was performed as previously described [25]. The histological sections of skin biopsies underwent deparaffinization in xylene and rehydration in water-ethanol solutions. The sections were then heated in a sodium citrate buffer (pH 6.0) for 30 min at 95 °C in a steam cooker, followed by a cooling period for sections to settle to room temperature. After rinsing the sections in PBS, a protein blocking buffer (Protein Block ab64226, Abcam, Cambridge, UK) was applied to the tissue for 20 min, to prevent non-specific staining. After another washing in PBS, the sections were incubated in a humid chamber overnight with primary antibodies (Table 1), followed by an additional rinsing in PBS. Then, appropriate secondary antibodies (Table 1) were applied and sections were incubated for an hour in a humid chamber. Afterward, sections were washed in PBS for a final time and DAPI (4′,6-diamidino-2-phenylindole) counterstaining for nuclei was applied for 2 min. Finally, the sections were rinsed with distilled water, air-dried for 15 min and cover-slipped (Immuno-Mount, Thermo Shandon, Pittsburgh, PA, USA). To prove the specificity of staining, control slides were made by omitting primary antibodies from the protocol, which resulted in the absence of staining of the tissue. The sections were examined using an Olympus fluorescence microscope (BX61; Tokyo, Japan) with a mounted digital camera (DP71). Microphotographs were captured using Nikon DS–Ri2 camera (Nikon Corporation, Tokyo, Japan) and plate assembly was performed using Adobe Photoshop v21.0.2. (Adobe, San Jose, CA, USA).

### 2.3. Epidermal Thickness and Inflammatory Infiltrate Density

Thickness of the cellular epidermis, which ranges from the basal layer to the beginning of the corneal layer, was measured using a modified version of the method described by Therkildsen et al. [26]. For each sample, we analyzed the epidermis at ×100 magnification and measured the shortest length from the dermo-epidermal junction (DEJ) to the corneal layer at ten representative areas selected after using the grid function of ImageJ software (NIH, Bethesda, MD, USA). The average of these lengths was used for further analysis. The density of the inflammatory infiltrates in LS samples was measured by using the freehand selection tool and the measure function of ImageJ to obtain the area that the infiltrate is present in. The value of the area was then divided by the length of the DEJ in the analyzed section to obtain infiltrate density.

### 2.4. Nerve Fiber Density

Intraepidermal nerve fiber density (IENFD) was calculated as described by the European Federation of Neurological Societies/Peripheral Nerve Society Guideline [27]. Briefly, three non-adjacent sections per sample were analyzed using a ×40 objective. Nerves stained with PGP 9.5 or CGRP that crossed the DEJ were counted, as well as nerves found intraepidermally without apparent crossing of the DEJ, if a corresponding subepidermal nerve was visible. Secondary branching of the nerves within the epidermis was not included in the calculation. Subepidermal nerve fiber density (SENFD) was calculated on the same sections as IENFD, using a modification of the method described by Vlcková-Moravcová et al. [28]. Individual nerve fibers found in the dermis within 50 µm of the DEJ were counted if they did not cross the DEJ or have a corresponding intraepidermal nerve fiber. The length of the DEJ was measured using ImageJ software (NIH, Bethesda, MD, USA). Results were expressed as the number of counted nerve fibers per mm of the DEJ.

### 2.5. Cell Densities and Proliferation Index

Mast cells and macrophages were counted on samples stained for tryptase and cd68, respectively. Three non-adjacent sections per sample were analyzed at ×100 magnification and all positively stained cells were counted after merging cell marker and DAPI staining using Adobe Photoshop. Additionally, we divided each analyzed section into a superficial region, containing the epidermis and dermis within 50 µm of the DEJ, and a deep region, which included the rest of the sample. Cell counts were performed for the individual regions. Cell densities were calculated as the ratio between the cell count and the length of the DEJ in the analyzed section. The proliferation index of the epidermis was calculated as the percentage of Ki-67-positive basal cells among all analyzed basal cells [29]. Cells were counted at ×100 magnification in three non-adjacent sections per sample.

### 2.6. Statistical Analysis

All data are expressed as the mean values of the groups of analyzed specimens. The normality of the distribution of the data was tested using the Shapiro–Wilk test. Statistical significance was determined by one-way ANOVA and Uncorrected Fisher’s LSD posthoc test for IENFD and CGRP-positive SENFD, while Welch ANOVA and Unpaired t with Welch’s correction posthoc test were used for PGP 9.5-positive SENFD. The statistical significance of differences in the age, epidermal thickness, proliferation index, mast cell, and macrophage densities was determined using the Kruskal–Wallis test with Uncorrected Dunn’s posthoc test. Mann–Whitney U test was used to test the significance of differences in inflammatory infiltrate density, time between disease onset and biopsy, and VAS score for itch. All analyses had *n* = 40 (4 groups of 10 samples). Statistical significance was set at *p* < 0.05.

## 3. Results

All the findings regarding morphology and protein expression described in our results were present in at least 80% of analyzed samples in their respective group. Representative images where the findings were the most evident were selected for the figures. The statistical analysis following descriptive findings gives insight into the average values of morphological and immunohistochemical features of the analyzed groups.

### 3.1. Clinical Characteristics of the Patients

The median age of all patients in the study was 63 years, with an interquartile range (IQR) of 57 to 70 years. There was no significant difference in the age of patients between the four analyzed groups. For patients with extragenital LS, the median time between disease onset and the initial clinical examination when the biopsy was performed was 2 months (IQR 1–3 months). In the case of genital LS patients, a median of 8 months (IQR 6–10 months) passed between disease onset and the biopsy procedure, which was significantly different from patients with extragenital LS (*p* < 0.001). VAS scores for the average level of itch were also significantly different between the two groups (*p* < 0.001). Patients with extragenital LS had a median VAS score of 0 (IQR 0–1), meaning no to very mild itch, while patients with genital LS had a median score of 8 (IQR 7–9), signifying extreme discomfort.

### 3.2. Hematoxylin and Eosin Staining

**Figure 1:**
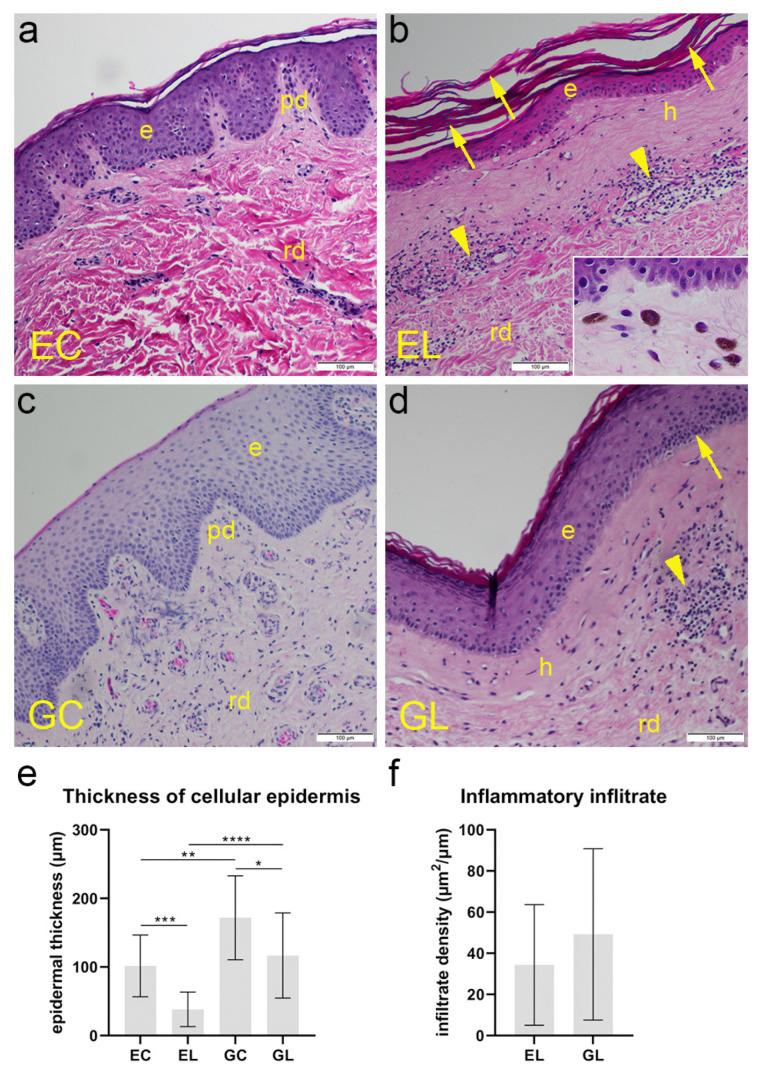
Morphology of normal skin and lichen sclerosus skin samples. EC—normal extragenital skin: typical histologic features of epidermis (e), papillary dermis (pd), and reticular dermis (rd) can be observed (**a**). EL—extragenital skin affected by lichen sclerosus: thinned epidermis (e) with hyperkeratosis (arrows), homogenization and hyalinization of the papillary dermis (h), and lymphocytic infiltrates (arrowheads) in the reticular dermis (rd) can be observed. An accumulation of melanophages is displayed in the inset (**b**). GC—normal vulvar skin: thick epidermis (e) with vascularized papillary (pd) and reticular dermis (rd) can be seen (**c**). GL—vulvar skin affected by lichen sclerosus: homogenization of the papillary dermis (h), a lymphocytic infiltrate (arrowhead) in the reticular dermis (rd), and lymphocyte exocytosis (arrow) in the thinned epidermis (e) can be seen (**d**). Hematoxylin and eosin staining, ×200 magnification; scale bars at the bottom right represent 100 µm. Analysis of epidermal thickness (**e**); statistical significance was determined by the Kruskal–Wallis test. Analysis of inflammatory infiltrate density measured as the ratio of the area the infiltrate is present in (µm^2^) and the length of the dermal-epidermal junction (**f**); statistical significance was determined by the Mann–Whitney U test. Graphs display mean values with standard deviations shown by error bars. The significance of differences is marked by asterisks: * *p* < 0.05, ** *p* < 0.01, *** *p* < 0.001, **** *p* < 0.001.

The normal skin consisted of two layers—epidermis and dermis. The epidermis (stratified squamous keratinized epithelium) had approximately 8–10 rows of epithelial cells—keratinocytes, with the thin surface layer of the flattened non-nucleated cells (squames). The wavy interface between the epidermis and dermis was formed by epidermal ridges and dermal papillae. The total number of keratinocyte rows was smaller at places where deep papillae penetrated the epidermis. The dermis contained the papillary and reticular layers. The papillary layer was more superficial and consisted of loose connective tissue with small blood vessels. Deeper, reticular layer contained irregular bundles of collagen fibers (Figure 1a). Compared to healthy skin, histologic sections of the skin with lichen sclerosus showed thinned epidermis with hyperkeratosis of the superficial layer, while in the basal layer, cells showed characteristic vacuolar degeneration. The epidermal-dermal interface appeared flattened and not wavy. The papillary dermis contained homogenized collagen, ectatic capillaries, and at places accumulated melanophages (inset of Figure 1b). Lymphocytic inflammatory infiltrate could be observed around the dilated small vessels (Figure 1b). The section of labia majora sample showed normal, relatively thick epidermis, with increased pigmentation of the basal layer due to higher skin pigmentation of the vulva. The dermo-epidermal junction was preserved, with very deep papillae and normal structure of dermal connective tissue, containing blood vessels and nerves (Figure 1c). Although the histopathological picture of vulvar LS can vary, the epidermis was thinned compared to healthy genital skin, showing more squames on the surface. Loss of rete pegs was accompanied by vacuolization of cells and flattened epidermal-dermal interface. The basement membrane was focally thickened, with homogenized and hyalinized collagen in the papillary layer. Dermal lymphocytic infiltrate was located below the hyalinized area, at the papillary-reticular border, but intraepidermal lymphocyte exocytosis was also present in some parts (Figure 1d). The average thickness of the cellular epidermis in EL samples was 38.2 µm, which was significantly lower (*p* < 0.001) than 101.6 µm in EC samples. The average thickness of GL samples (116.7 µm) was also significantly lower (*p* = 0.026) than in GC samples (171.8 µm). There was also a significant difference in epidermal thickness between sample locations, with extragenital epidermis being significantly thinner compared to genital epidermis, both between healthy (*p* = 0.003) and LS (*p* < 0.001) samples (Figure 1e). While the density of the inflammatory infiltrate was higher in GL samples (49.18 µm^2^ per µm of the DEJ) compared to EL samples (34.36 µm^2^/µm), the difference was not statistically significant (Figure 1f).

### 3.3. Double Immunofluorescence Staining to VR1 and CGRP

**Figure 2:**
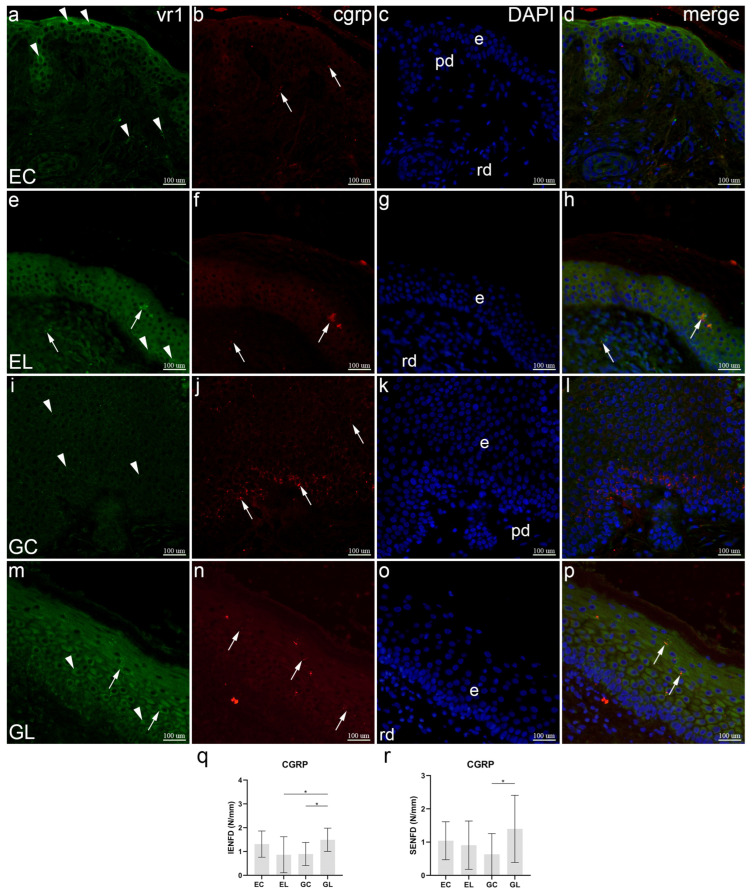
Co-expression of VR1 and CGRP in normal skin and lichen sclerosus skin samples. The epidermis (e), papillary dermis (pd), and reticular dermis (rd) are marked on DAPI staining images which display all cell nuclei (**c**,**g**,**k**,**o**). EC—normal extragenital skin (**a**–**d**): VR1 is expressed in several keratinocytes and dermal cells (arrowheads, (**a**)); CGRP expression is observed in nerve fibers (arrows, (**b**)); there is no co-expression of VR1 and CGRP (**d**). EL—extragenital skin affected by lichen sclerosus (**e**–**h**): VR1 is expressed in keratinocytes (arrowheads) and nerve fibers (arrows), both intra- and subepidermal (**e**); CGRP is expressed in nerve fibers (arrows, (**f**)); VR1 and CGRP are co-expressed in nerve fibers (arrows, (**h**)). The epidermis of this sample is thickened, which is uncommon for extragenital LS, however, other features of LS, like homogenization of the papillary dermis, are present. GC—normal vulvar skin (**i**–**l**): VR1 expression is seen in keratinocytes (arrowheads, (**i**)); CGRP stains nerve fibers (arrows, (**j**)); VR1 and CGRP are not co-expressed (**l**). GL—vulvar skin affected by lichen sclerosus (**m**–**p**): keratinocytes (arrowheads) and intraepidermal nerve fibers (arrows) express VR1 (**m**); intraepidermal nerve fibers express CGRP (arrows, (**n**)); co-expression of VR1 and CGRP is present in nerve fibers (arrows, (**p**)). Double immunofluorescence staining to VR1, CGRP and DAPI, ×400 magnification. Analysis of CGRP-positive intraepidermal nerve fiber density (IENFD) (**q**); statistical significance was determined by one-way ANOVA. Analysis of CGRP-positive subepidermal nerve fiber density (SENFD) (**r**); statistical significance was determined by one-way ANOVA. N/mm denotes the number of nerve fibers per mm of dermal-epidermal junction. Graphs display mean values with standard deviations shown by error bars. The significance of differences is marked by asterisks: * *p* < 0.05.

The epidermis of the control skin samples (EC) showed immunoreactivity to vanilloid receptor subtype 1 (VR1) in some cells of the hair follicle, and intense reactivity in the superficial keratinocytes. VR1 was present only weakly in a few dermal cells. CGRP-positive nerve fibers were mostly present in the dermis and were occasionally seen between basal cells of the epidermis. DAPI nuclear stain characterized all cell nuclei. Merging of the three stainings revealed that VR1 did not co-express with CGRP in the same cells or fibers (Figure 2a–d). Compared to control healthy skin, in extragenital lichen sclerosus, VR1 was moderately expressed in epidermal suprabasal cells, weakly in the dermal nerve fibers and strongly in the nerve fibers of superficial epidermal layers. Opposite to healthy skin, CGRP strongly stained nerve fibers in the epidermis, and only occasionally in the dermis. While co-expression was missing in the healthy skin, here the co-expression of VR1/CGRP was observed in epidermal and some dermal nerve fibers, but not in the epithelial skin cells (Figure 2e–h). Normal genital skin of the vulva showed weak to moderate expression of VR1 in some epidermal basal and spinous cells. Compared to extragenital skin, CGRP was strongly expressed in nerve fibers that passed between the basal cells of the epidermis, while only occasionally in the suprabasal and spinous epidermal layers. There was no co-expression of the used markers (Figure 2i–l). Compared to healthy genital skin, VR1 expression in patients with genital lichen sclerosus was strong in the keratinocytes of all epidermal layers and in intraepidermal nerve fibers. Strong expression of CGRP characterized suprabasal epithelium. In contrast to control sample, co-expression of the two markers was observed in nerve fibers of the epidermis (Figure 2m–p). Mean IENFD for CGRP-positive fibers was 1.49 IENF/mm in GL samples, which was significantly higher compared to 0.9 IENF/mm in GC samples (*p* = 0.028) and 0.86 IENF/mm in EL samples (*p* = 0.021). There were no other significant differences between the studied groups (Figure 2q). Analysis of SENFD showed a significant difference between GL samples with a mean of 1.4 SENF/mm and GC samples with 0.63 SENF/mm (*p* = 0.028), while there were no other significant differences between the analyzed groups (Figure 2r).

### 3.4. Double Immunofluorescence Staining to VR1 and PGP 9.5

**Figure 3:**
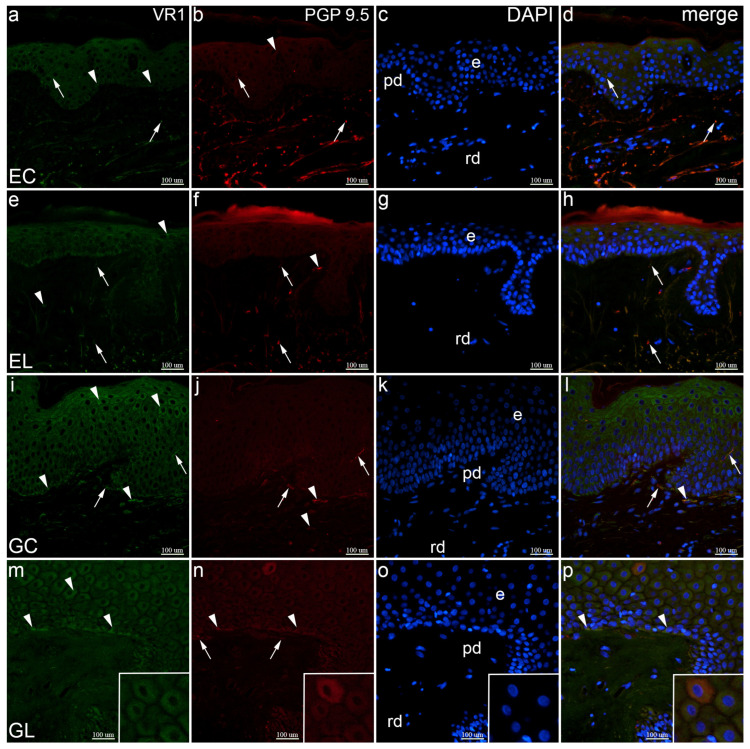
Co-expression of VR1 and PGP 9.5 in normal skin and lichen sclerosus skin samples. The epidermis (e), papillary dermis (pd), and reticular dermis (rd) are marked on DAPI staining images which display all cell nuclei (**c**,**g**,**k**,**o**). EC—normal extragenital skin (**a**–**d**): keratinocytes (arrowheads) and nerve fibers (arrows) display VR1 expression (**a**); keratinocytes (arrowhead) and nerve fibers (arrows) also express PGP 9.5 (**b**); VR1 and PGP 9.5 are co-expressed in nerve fibers (arrows, (**d**)). EL—extragenital skin affected by lichen sclerosus (**e**–**h**): keratinocytes and some dermal cells (arrowheads) as well as nerve fibers (arrows) show VR1 expression (**e**); dermal cells (arrowhead) and nerve fibers (arrows) are PGP 9.5-positive (**f**); VR1 and PGP 9.5 are co-expressed in nerve fibers (arrows, (**h**)). GC—normal vulvar skin (**i**–**l**): keratinocytes and some dermal cells (arrowheads), and nerve fibers (arrows) show VR1 expression (**i**); dermal cells (arrowheads) and nerve fibers (arrows) also express PGP 9.5 (**j**); nerve fibers (arrows) and a dermal cell (arrowhead) display VR1 and PGP 9.5 co-expression (**l**). GL—vulvar skin affected by lichen sclerosus (**m**–**p**): VR1 is expressed in keratinocytes (arrowheads, (**m**)); keratinocytes (arrowheads) and nerve fibers (arrows) express PGP 9.5 (**n**); co-expression of VR1 and PGP 9.5 is seen in keratinocytes (arrowheads, (**p**)); insets display VR1 expression (**m**) and strong PGP 9.5 expression (**n**) which co-localize in a keratinocyte (**p**). Double immunofluorescence staining to VR1, PGP 9.5 and DAPI, ×400 magnification.

In the section of normal extragenital skin, VR1 showed moderate expression throughout the epidermis, while weak expression was observed in the intraepidermal and deeper dermal nerve fibers. PGP 9.5 strongly stained nerve fibers, in superficial and deep dermal layers, and only a few cells in the epidermis. Co-localization of VR1 and PGP 9.5 was seen in intraepidermal and dermal nerve fibers (Figure 3a–d). Compared to the control samples, VR1 expression was moderate in basal cells and some spinous cells, while in the dermis its expression was moderate. PGP 9.5 showed moderate expression in the dermal nerve fibers, and some dermal cells, while its expression was missing in the epidermis. Their co-localization characterized only the dermal nerve fibers, while it was missing intra-epidermally (Figure 3e–h). Moderate to strong immunoreactivity of VR1 was observed throughout the vulvar epidermis, especially in the superficial layer, and in the papillary dermis. PGP 9.5 showed strong expression in the horizontally oriented fibers of the papillary dermis and in the intraepidermal nerve fibers. Their co-expression was seen in some horizontal dermal nerve fibers, Schwann cells, and some intraepidermal nerve fibers (Figure 3i–l). Compared to the control samples, in genital LS, suprabasal cells and a few basal cells showed moderate expression of VR1, while the dermis showed no positivity. Moderate to strong PGP 9.5 expression characterized basal epidermal cells and small fibers on the dermo-epidermal junction. Merging of all markers revealed co-expression of VR1 and PGP 9.5 in basal cells, but in contrast to control tissue, not in the nerve fibers (Figure 3m–p).

### 3.5. Double Immunofluorescence Staining to VR1 and Tryptase

**Figure 4:**
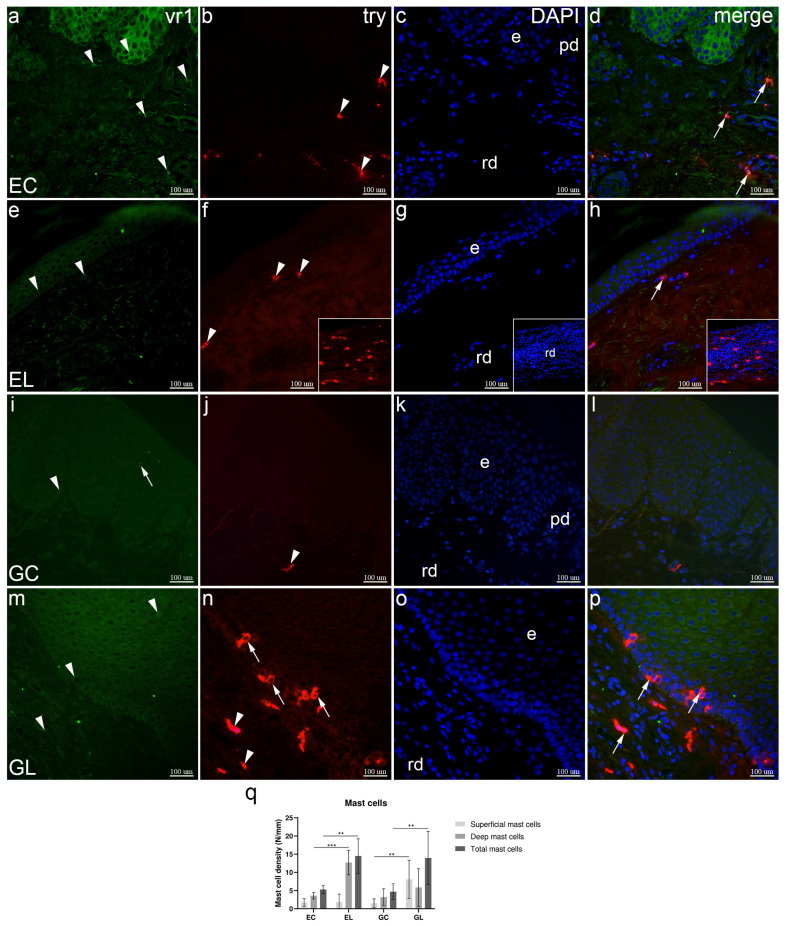
Co-expression of VR1 and tryptase (try) in normal skin and lichen sclerosus skin samples. The epidermis (e), papillary dermis (pd), and reticular dermis (rd) are marked on DAPI staining images which display all cell nuclei (**c**,**g**,**k**,**o**). EC—normal extragenital skin (**a**–**d**): VR1 expression is observed in keratinocytes and dermal cells (arrowheads, (**a**)); tryptase staining displays dermal mast cells (arrowheads, (**b**)); VR1 and tryptase are co-expressed in mast cells (arrows, (**d**)). EL—extragenital skin affected by lichen sclerosus (**e**–**h**): keratinocytes and dermal cells show VR1 expression (arrowheads, (**e**)); dermal mast cells express tryptase (arrowheads, (**f**)); co-expression of VR1 and tryptase is observed in a mast cell (arrow, (**h**)); insets demonstrate a mast cell infiltrate in the reticular dermis (**f**–**h**). GC—normal vulvar skin (**i**–**l**): keratinocytes (arrowhead) and a nerve fiber (arrow) show VR1 expression (**i**); a dermal cell shows tryptase staining (arrowhead, (**j**)); no co-expression is observed in the merged image (**l**). GL—vulvar skin affected by lichen sclerosus (**m**–**p**): VR1 is expressed in keratinocytes and dermal cells (arrowheads, (**m**)); dermal (arrowheads) and intraepidermal (arrows) mast cells are displayed by tryptase staining (**n**); co-expression of VR1 and tryptase is seen in mast cells (arrows, (**p**)). Double immunofluorescence staining to VR1, tryptase and DAPI, ×400 magnification. Analysis of mast cell density (**q**); statistical significance was determined by the Kruskal–Wallis test. N/mm denotes the number of mast cells per mm of dermal-epidermal junction. Graphs display mean values with standard deviations shown by error bars. The significance of differences is marked by asterisks: ** *p* < 0.01, *** *p* < 0.001.

Healthy extragenital skin showed moderate to strong expression of VR1 in the epidermal cells, and moderate expression in dermal cells. Tryptase (try) staining demonstrated strong expression in certain dermal cells (mast cells), mostly in the deeper dermis. Colocalization of VR1 and try was present in mast cells (Figure 4a–d). Compared to healthy skin, in the tissue sample of patients with extragenital LS, VR1 was weaker in epidermal and some dermal cells. Try staining revealed mast cells in the superficial layer of the dermis and in the infiltrate in the deeper dermis. VR1 and tryptase co-localized in the mast cells (Figure 4e–h). Compared to healthy extragenital skin, weaker VR1 staining was observed in most of the epidermis and in intraepidermal nerve fibers of the vulvar control sample. Tryptase stained mast cells in the dermis. There was no co-localization of VR1 and try in the overlapped images (Figure 4i–l). Most of the keratinocytes and some dermal cells showed moderate expression of VR1, while try staining demonstrated several dermal and many intraepidermal mast cells inside the basal layer. In contrast to healthy vulvar skin, co-localization of VR1 and try staining characterized mast cells. (Figure 4m–p). The median total mast cell density in EL samples was 14.50 cells/mm, which was significantly higher than 5.30 cells/mm in EC samples (*p* = 0.001). The same was found for GL samples with 13.95 cells/mm, compared to GC samples with 4.69 cells/mm (*p* = 0.001). When analyzing the superficial and deeper regions of the dermis separately, we found differences between sites. The median mast cell density of the superficial region in GL samples equaled 8.10 cells/mm and was significantly higher compared to 1.49 cells/mm in GC samples (*p* = 0.003). There was no significant difference between EL and EC samples in the superficial region. The median mast cell density of the deeper region in EL samples was 12.70 cells/mm, which was significantly higher than 3.59 cells/mm in EC samples (*p* < 0.001). There was no significant difference between GL and GC samples in the deeper region (Figure 4q).

### 3.6. Double Immunofluorescence Staining to Tryptase and Ki-67

**Figure 5:**
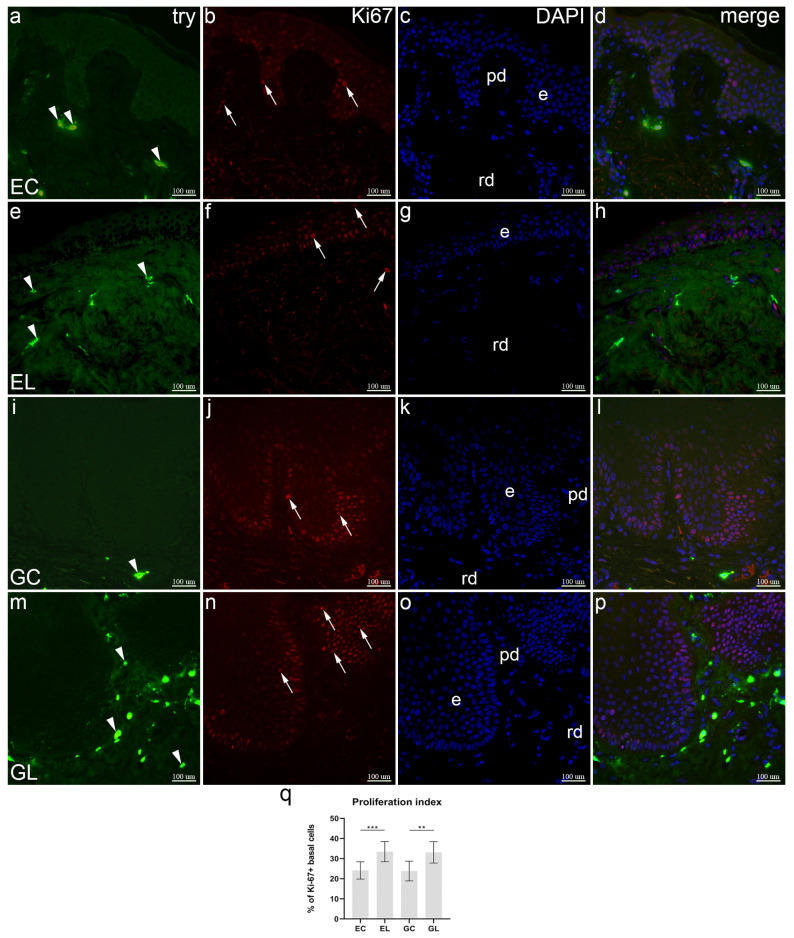
Co-expression of tryptase (try) and Ki-67 in normal skin and lichen sclerosus skin samples. The epidermis (e), papillary dermis (pd), and reticular dermis (rd) are marked on DAPI staining images which display all cell nuclei (**c**,**g**,**k**,**o**). EC—normal extragenital skin (**a**–**d**): tryptase expression is observed in dermal mast cells (arrowheads, (**a**)); nuclear Ki-67 expression demonstrates proliferating cells in the dermis and epidermis (arrows, (**b**)); nuclear Ki-67 staining is not present in tryptase-positive mast cells (**d**). EL—extragenital skin affected by lichen sclerosus (**e**–**h**): dermal mast cells display tryptase expression (arrowheads, (**e**)); proliferating cells of the dermis and epidermis show nuclear Ki-67 staining (arrow, (**f**)); co-expression of nuclear Ki-67 and tryptase is not observed (**h**). GC—normal vulvar skin (**i**–**l**): tryptase staining reveals a mast cell (arrowhead, (**i**)); epidermal cell proliferation is displayed by nuclear Ki-67 staining (arrows, (**j**)); no co-expression is observed in the merged image (**l**). GL—vulvar skin affected by lichen sclerosus (**m**–**p**): many dermal mast cells are demonstrated by tryptase staining (arrowheads, (**m**)); extensive proliferation of keratinocytes is displayed by Ki-67 staining (arrows, (**n**)); dermal mast cell do not co-express Ki-67 (**p**). Double immunofluorescence staining to tryptase, Ki-67 and DAPI, ×400 magnification. Analysis of epidermal proliferation index (**q**); statistical significance was determined by the Kruskal–Wallis test. Graphs display mean values with standard deviations shown by error bars. The significance of differences is marked by asterisks: ** *p* < 0.01, *** *p* < 0.001.

Normal skin showed strong try staining in several mast cells located primarily in the papillary dermis. Strong Ki-67 staining was restricted to the basal and suprabasal keratinocytes and some cells in the dermis. Mast cells did not co-localize with proliferating Ki-67 positive cells (Figure 5a–d). Compared to the control samples, several mast cells were try-positive in the dermis, while Ki-67 positive cells were seen both in the epidermis and dermis. No co-expression of Ki-67 and try positive cells was present (Figure 5e–h). Tryptase staining demonstrated a couple of mast cells in the dermis of healthy vulvar skin, while keratinocytes of the basal layer and some regions of the suprabasal layer showed strong Ki-67 staining. No mast cells co-localized with Ki-67 positive proliferating cells (Figure 5i–l). Compared to control vulvar skin, a large number of mast cells was revealed by tryptase staining in the subepithelial part (papillary layer) of the dermis, and a few in the deeper dermis. Strong Ki-67 expression characterized the basal and suprabasal epidermal layers. Try positive mast cells did not co-localize with Ki-67 positive cells (Figure 5m–p). The median epidermal proliferation index of both EL (33.07%) and GL (31.58%) was significantly higher compared to their controls—24.8% for EC and 24% for GC (*p* < 0.001, *p* = 0.002, respectively). There were no differences in the proliferation index in regard to the site (genital vs. extragenital), both for lichen and control samples (Figure 5q).

### 3.7. Immunofluorescence Staining to PGP 9.5

**Figure 6:**
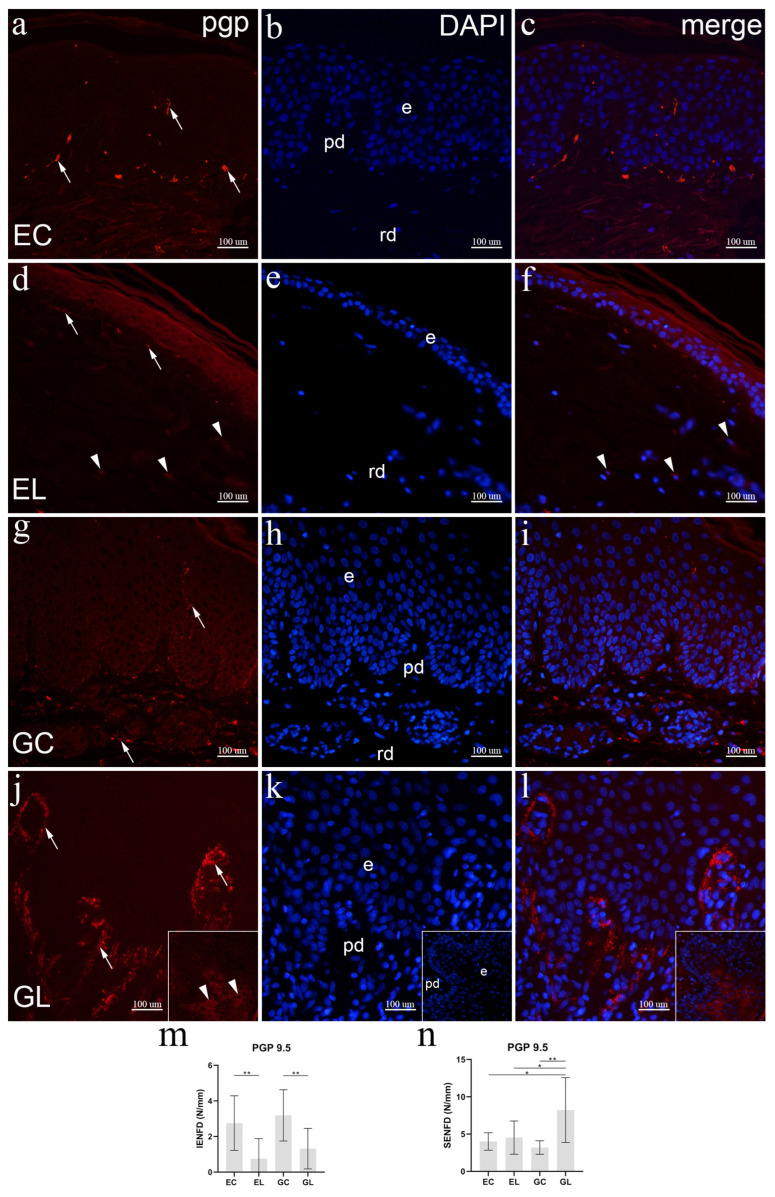
Expression of PGP 9.5 in normal skin and lichen sclerosus skin samples. The epidermis (e), papillary dermis (pd), and reticular dermis (rd) are marked on DAPI staining images which display all cell nuclei (**b**,**e**,**h**,**k**). EC—normal extragenital skin (**a**–**c**): PGP 9.5 expression is present in intraepidermal and subepidermal nerve fibers (arrows, (**a**)). EL—extragenital skin affected by lichen sclerosus (**d**–**f**): subepidermal nerve fibers (arrows) and some dermal cells (arrowheads) show PGP 9.5 expression (**d**); cytoplasmatic PGP 9.5 expression is seen in dermal cells (arrowheads, (**f**)). GC—normal vulvar skin (**g**–**i**): intraepidermal and subepidermal nerve fibers show PGP 9.5 expression (arrows, (**g**)). GL—vulvar skin affected by lichen sclerosus (**j**–**l**): strong PGP 9.5 expression is present in multiple subepidermal nerve fibers (arrows, (**j**)); insets display moderate PGP 9.5 expression in suprabasal keratinocytes (arrowheads) of the epiderms (**j**–**l**). Immunofluorescence staining to PGP 9.5 and DAPI, ×400 magnification. Analysis of PGP 9.5-positive intraepidermal nerve fiber density (IENFD) (**m**); statistical significance was determined by one-way ANOVA. Analysis of PGP 9.5-positive subepidermal nerve fiber density (SENFD) (**n**); statistical significance was determined by Welch ANOVA. N/mm denotes the number of nerve fibers per mm of dermal-epidermal junction. Graphs display mean values with standard deviations shown by error bars. The significance of differences is marked by asterisks: * *p* < 0.05, ** *p* < 0.01.

Healthy extragenital skin contained many PGP 9.5-positive nerve fibers in the papillary dermis, while they were less extensive in the epidermis and the deeper parts of the dermis (Figure 6a–c). Compared to healthy skin, fewer nerve fibers showing moderate to strong PGP 9.5 staining were visible in the papillary dermis, occasionally in the deeper dermis, while no nerve fibers were present in the epidermis (Figure 6d–f). Normal vulvar skin revealed multiple PGP 9.5 positive nerve fibers both in the subepidermal and deeper parts of the dermis, as well as in the epithelium, reaching the superficial parts of the spinous layer. All the nerve fibers showed strong PGP 9.5 staining (Figure 6g–i). Compared to control vulvar skin, a large amount of strongly PGP 9.5 staining nerve fibers accumulated in the papillary dermis, while they were absent in the epidermis (Figure 6j–l). The average IENFD of PGP 9.5 positive fibers in EL samples equaled 0.75 IENF/mm, which compared to 2.75 IENF/mm in EC samples was significantly lower (*p* = 0.002). The average values of IENFD in GL (1.32 IENF/mm) and GC samples (3.19 IENF/mm) were also significantly different (*p* = 0.003). There was no difference between genital and extragenital sites, both for lesions and controls (Figure 6m). The SENFD of GL samples (8.22 SENF/mm), compared to GC (3.2 SENF/mm), EL (4.53 SENF/mm), and EC samples (4.01 SENF/mm) was significantly higher (*p* = 0.005, *p* = 0.032, *p* = 0.014, respectively). There was no significant difference between the other groups of samples (Figure 6n).

### 3.8. Immunofluorescence Staining to CD68

**Figure 7:**
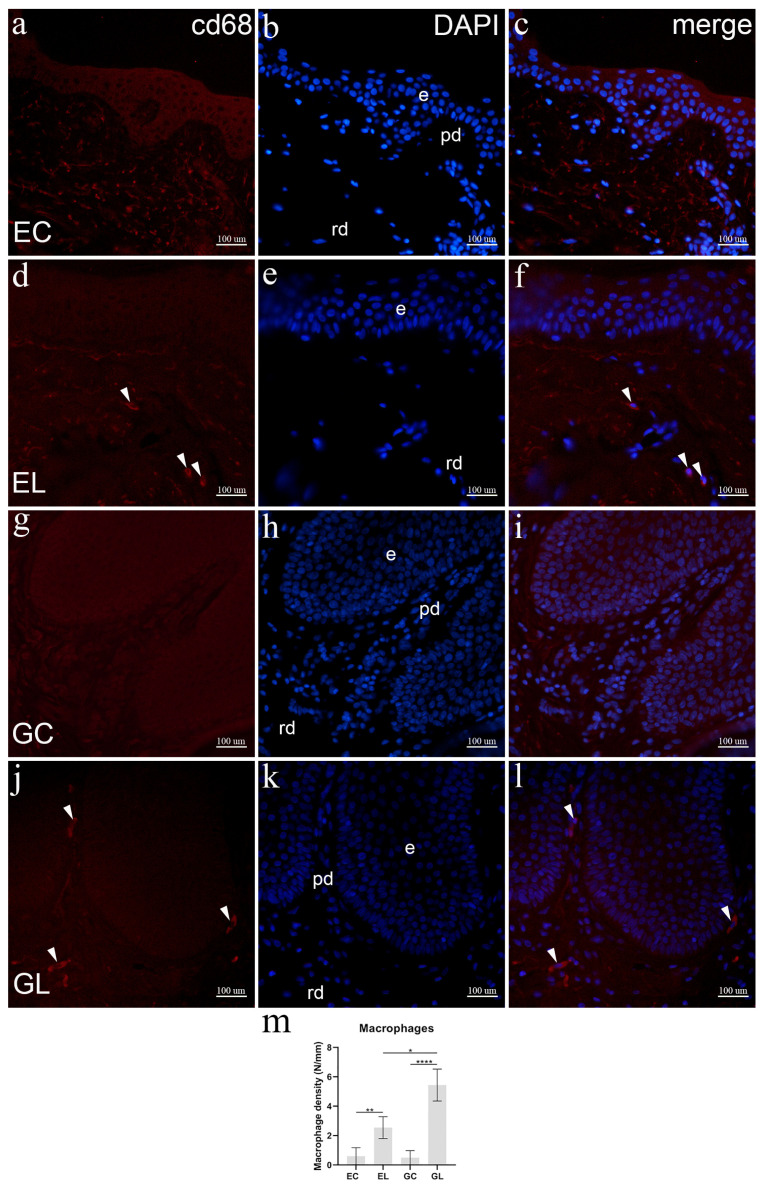
Expression of CD68 in normal skin and lichen sclerosus skin samples. The epidermis (e), papillary dermis (pd), and reticular dermis (rd) are marked on DAPI staining images which display all cell nuclei (**b**,**e**,**h**,**k**). EC—normal extragenital skin (**a**–**c**): no CD68 positive cells are displayed. EL—extragenital skin affected by lichen sclerosus (**d**–**f**): several macrophages are demonstrated by CD68 expression (arrowheads, (**d**,**f**)). GC—normal vulvar skin (**g**–**i**): CD68 expression is not observed in dermal cells. GL—vulvar skin affected by lichen sclerosus (**j**–**l**): CD68-positive macrophages are seen in the dermis (arrowheads, (**j**,**l**)). Immunofluorescence staining to CD68 and DAPI, ×400 magnification. Analysis of macrophage cell density (**m**); statistical significance was determined by the Kruskal–Wallis test. N/mm denotes the number of macrophages per mm of dermal-epidermal junction. Graphs display mean values with standard deviations shown by error bars. The significance of differences is marked by asterisks: * *p* < 0.05, ** *p* < 0.01, **** *p* < 0.001.

In control skin samples, no CD68 staining was visible in the papillary and reticular dermis. Multiple elastic fibers in the dermis showed autofluorescence, which may mimic the CD68 signal. Some macrophages could be seen in the deeper layers of the reticular dermis (not shown) (Figure 7a–c). Compared to healthy skin, LS samples showed CD68-postive macrophages in the superficial papillary layer and in the deeper part of the dermis (Figure 7d–f). There were no CD68-positive cells present in the papillary or superficial part of the reticular dermis in the analyzed samples of healthy vulvar skin (Figure 7g–i). Compared to healthy vulvar skin, macrophages stained positively for CD68 were present in the superficial papillary dermis, often near the dermo-epidermal junction (Figure 7j–l). The median macrophage density in EL samples was 2.54 cells/mm, which was significantly higher compared to 0.60 cells/mm in EC samples (*p* = 0.009). This was also found when comparing GL samples, having 5.44 cells/mm, with GC samples containing a median of 0.50 cells/mm (*p* < 0.001). There was a statistically significant difference in macrophage density between EL and GL samples (*p* = 0.049); however, there was no significant difference between EC and GC samples (Figure 7m).

## 4. Discussion

Our study reports on the altered distribution of CGRP and VR1 in the nerve fibers and cells of extragenital and vulvar skin affected by lichen sclerosus, as well as the mast cell and macrophage infiltrates present in the same tissues. Previously, the altered distribution of CGRP-positive fibers in vulvar LS and the distribution of VR1 in healthy and pruritic skin was described [30,31]. For other pruritic dermatoses, like psoriasis and atopic dermatitis, neurogenic inflammation mediated by CGRP-positive nerve fibers, VR1, and mast cells is a major component of their pathogenesis [9]. Taking this into account, we suggest that neurogenic inflammation is one of the main pathogenetic mechanisms of lichen sclerosus.

We found no difference in the ages of the patients between the analyzed groups, meaning LS patients were appropriately matched with controls and age was not a confounding factor. We explain the difference in the time from disease onset to the first dermatologic examination (when biopsies were taken) between extragenital and genital LS by the fact that all patients with genital LS reported to their gynecologist first and were later referred to a dermatologist, while patients with extragenital LS directly went to a dermatologist. Another possible factor for this difference could be the reluctancy of women to seek treatment for vulvovaginal issues [32]. Our patients with genital LS suffered from severe itch on average, while patients with extragenital LS had no or very mild itch, which is in line with previously reported data [1,6].

There is a lack of histological diagnostic criteria for different stages and subtypes of lichen sclerosus [33]. In our study, we found decreased epidermal thickness in LS samples compared to their respective controls, with genital LS samples having significantly thicker epidermis than controls, which is in line with previous studies [8]. There was no significant difference in the amount of inflammatory infiltrate between genital and extragenital LS samples in our study, however, the distribution of the infiltrate and the presence of intraepithelial lymphocytes that we described has been shown to be typical for early LS lesions [34].

All cutaneous nerve fibers are visualized by PGP 9.5/UCHL 1, a generally applicable marker of nerves and neuroendocrine cells in vertebrates [14]. Similar to previous studies, our study showed fever PGP 9.5 positive intraepidermal nerve fibers in vulvar LS tissues compared to controls [30], which was suggested to be due to a chronic inflammatory autoimmune process. There was no difference in the expression of this neuropeptide between genital and extragenital LS tissues. However, analyzing only subepidermal nerve fibers, there was a statistically significant higher expression of PGP 9.5-positive fibers in genital LS tissues compared to extragenital LS and both controls, and that can be one of the contributing factors as to why this form of the disease is the most pruritic.

Calcitonin gene-related peptide is the main neurotransmitter of the nociceptive sensory C-fibers and its main function is vasodilatation and activation of mast cells [35]. In pruritic dermatoses, there is a higher expression of CGRP, as pruritic sensation can be caused by impairment of nerve fibers [19]. In our study, we found a higher number of CGRP-positive intraepidermal nerve fibers in vulvar LS compared to extragenital LS and vulvar controls, while there were no significant differences between extragenital lichen tissue and extragenital controls. Analysis of subepidermal CGRP-positive fibers revealed higher expression in vulvar, but not extragenital LS, compared to their respective controls. These findings could be a potential cause of more exaggerated pruritus that is normally found in vulvar cases. Many studies have also demonstrated that CGRP regulates various inflammatory processes in human skin [35]. Hyperinnervation of the epidermis by CGRP-positive fibers has been described in different dermatoses, including vulvar LS [30].

It is known that the expression of non-selective cation channels that participate in nociception and cutaneous neurogenic inflammation (TRPV1 and TRPA1) is not only seen in sensory nerves of the skin, but also in mast cells, dendritic cells, endothelial cells, and keratinocytes [10]. In other pruritic dermatoses, such as psoriasis and atopic dermatitis, increased TRPV1 expression on nerve fibers and subsequent release of CGRP are the mainstream causes of the vicious pro-itch cytokine cycle [9]. Our study of TRPV1 expression found no differences in the distribution of TRPV1 among keratinocytes and mast cells in the investigated groups of samples. However, our analysis of TRPV1 in nerve fibers revealed expression in CGRP-positive nerve fibers in both the vulvar and extragenital LS samples, while there was no expression of the same types of fibers in the control groups. This was true for both intraepidermal and subepidermal CGRP-positive fibers. In skin inflammatory conditions, the number of TRPV1 receptor-positive nonneural cells is increased [36]. Taking all of this into account, TRPV1 could have a role in the pathogenesis of lichen sclerosus [30].

In addition to changes in nerve fibers, mast cells have been found to have important roles in the pathogenesis of pruritic diseases [18]. In normal skin, mast cell numbers are highest in the upper dermis, regardless of sex and age [19]. An increased number of mast cells was found in the upper parts of the dermis in pruritic skin diseases like psoriatic lesions [37] and lesional atopic dermatitis [38]. We have also observed a higher number of mast cells in both vulvar and extragenital LS samples compared to their respective control sites. However, the distribution of mast cells was different between the two sites: in the vulvar LS, the increased number of mast cells was primarily concentrated in the superficial parts of the dermis and even intraepithelially, while in the extragenital LS the increase in number was mainly attributed to the mast cells in the deeper dermis. Analysis of the proliferation marker Ki-67 revealed that mast cells do not proliferate, which indicates that the increase in their numbers in LS samples could be caused by the increased mast cell migration into these lesions [39]. The different distribution of mast cells between the two types of LS could be one possible explanation for the difference in pruritic symptoms between them.

Macrophages are a type of cell whose number is increased in the inflammatory infiltrate in LS [40], and they are usually present in the band of inflammatory cells or scattered in the sclerotic region [21]. In our study, we have also observed a higher number of macrophages in extragenital and genital lichen samples, compared to control samples. The distribution of macrophages in our extragenital and genital samples was similar to those observed in the studies so far [21]. Macrophages are normally found in tissues with chronic inflammation [41], and their M2 subtype can contribute to tissue fibrosis [42]. M2 macrophages have also been shown to contribute to pruritus via IL-31 in some dermatoses [43], but we have not found any studies linking pruritus in LS to macrophages. We have described a higher number of macrophages in vulvar LS, compared to extragenital LS samples, which may contribute to the difference in pruritic symptoms. However, additional studies on macrophages and IL-31 in LS are needed to draw any meaningful conclusions.

The proliferation of keratinocytes in LS has already been studied by a group of authors that have demonstrated that the proliferation marker Ki-67 is significantly up-regulated in vulvar LS compared to controls and its extragenital counterpart [20]. In our study, we found a significantly higher proliferation index, measured by the number of Ki-67+ basal cells, in both vulvar LS and extragenital LS, compared to their control sites. However, we have not found a significant difference between the two groups of LS samples. The increased proliferation of keratinocytes in LS could be the result of multiple factors. The direct stimulatory effect of CGRP on keratinocyte proliferation via activation of MAP kinases has previously been described [44], and we have also demonstrated an increased number of CGRP-positive nerve fibers in vulvar LS. It has been shown that proliferation of keratinocytes could be induced by an increased number of mast cells, especially near the dermo-epidermal junction [45], as is the case in vulvar LS. Interestingly, another study suggested that tryptase could have an inhibitory effect on keratinocyte proliferation by decreasing responses to epidermal growth factor upon costimulation [46]. Additionally, it was demonstrated that CGRP could induce epithelial cell proliferation mediated by mast cells via transforming growth factor-beta [47]. Yet, another possible cause of increased keratinocyte proliferation could be a repeated mechanical irritation of the pruritic skin due to scratching, which is typical for vulvar LS [48,49,50]. All of the above-mentioned inducers of keratinocyte proliferation (CGRP, subepidermal mast cells, mechanical irritation) were significantly higher in vulvar LS compared to extragenital LS, but there was no significant difference in the proliferation index. This can perhaps be explained by the methodology we used to measure keratinocyte proliferation in this study. We only analyzed keratinocytes in the basal layer [29] and indeed, there was no significant difference between the LS groups. However, suprabasal keratinocytes also proliferate and we have even described keratinocyte proliferation in the more superficial layers of the epidermis in LS samples. Since the epidermis in vulvar LS is markedly thicker than in extragenital LS, the total number of proliferating keratinocytes may be higher in the vulvar LS samples.

Some of the histopathological differences between the LS groups could be a consequence of the difference in their clinical characteristics. The time from disease onset to biopsy was longer for genital LS lesions, giving them more time to develop. Genital LS lesions were also characterized by severe itch and the mechanical irritation of the lesions from the accompanying scratching could also contribute to the difference in their histopathological features [49], since extragenital lesions do not cause significant itch [1,6].

Our findings provide potential targets for the research and development of novel anti-inflammatory and/or antipruritic therapeutic strategies which could not only alleviate symptoms, but also reduce reoccurrence and progression of the disease. The currently used first-line therapy option, topical glucocorticoids, have been implied to have a beneficial effect on neurogenic inflammation by lowering CGRP expression in sensory neurons [51], however, other studies have found that glucocorticoids can have differential effects on CGRP levels depending on cell type [52]. Regarding VR1, glucocorticoids have been shown to be able to both decrease [53] and increase its expression [54]. Pimecrolimus, a topical calcineurin inhibitor, has been shown to stimulate the release of CGRP from nerve fibers and activate VR1 [55], with the long-term effect of desensitizing VR1 and alleviating itch [56]. Additionally, it was found that calcineurin inhibitors are neurotrophic and stimulate the growth of CGRP-positive nerve fibers [57]. Glucocorticoids and calcineurin inhibitors both prevent mast cell activation and reduce mast cell numbers via activation of apoptosis [58,59].

In conclusion, we have found differences in VR1 distribution and keratinocyte proliferation between healthy skin and LS-affected skin. We have also found differences in innervation, mast cell and macrophage number and distribution between healthy and LS skin, but also between vulvar and extragenital LS sites. These differences could be a contributing factor to the contrasting pruritic symptoms of the individual sites and indicate that extragenital and genital LS, which are considered to be two clinical variants of the same disease, have different underlying histopathologic changes.

## Figures and Tables

**Table 1:** Primary and secondary antibodies used in the study.

	Antibodies	Host	Code No.	Dilution	Source
Primary	Anti-CD68	Mouse	ab31630	1:150	Abcam (Cambridge, UK)
	Anti-Ki-67	Rabbit	AB9260	1:300	EMD Millipore (Temecula, CA, USA)
	Anti-tryptase	Mouse	ab2378	1:500	Abcam (Cambridge, UK)
	anti-VR1	Rabbit	ab3487	1:100	Abcam (Cambridge, UK)
	anti-PGP9.5	Mouse	480012	1:500	Invitrogen (Camarillo, CA, USA)
	anti-CGRP	Goat	ab36001	1:400	Abcam (Cambridge, UK)
Secondary	Alexa Fluor^®^488 Anti-Mouse lgG (H + L)	Donkey	715-545-150	1:400	Jackson Immuno Research Laboratories (Baltimore, PA, USA)
	Alexa Fluor^®^488 Anti-Goat lgG (H + L)	Donkey	705-545-003	1:400	Jackson Immuno Research Laboratories (Baltimore, PA, USA)
	Alexa Fluor^®^488 Anti-Rabbit lgG (H + L)	Donkey	711-545-152	1:400	Jackson Immuno Research Laboratories (Baltimore, PA, USA)
	Rhodamine Red™-X Anti-Goat IgG (H + L)	Donkey	705-295-003	1:400	Jackson Immuno Research Laboratories (Baltimore, PA, USA)
	Rhodamine Red™-X Anti-Mouse IgG (H + L)	Donkey	715-295-151	1:400	Jackson Immuno Research Laboratories (Baltimore, PA, USA)

## Data Availability

The data presented in this study are available from the corresponding author upon reasonable request.
